# Autonomic dysfunction in patients with wild-type transthyretin amyloidosis

**DOI:** 10.1007/s00415-025-13604-0

**Published:** 2026-02-27

**Authors:** Vera E. A. Kleinveld, Julia Wanschitz, Anna Hotter, Corinne G. C. Horlings, Fabian Leys, Maria Ungericht, Gerhard Pölzl, Roberta Granata, Alessandra Fanciulli, Wolfgang N. Löscher

**Affiliations:** 1 Department of Neurology, Medical University of Innsbruck, Innsbruck, Austria; 2 Department of Cardiology, Medical University of Innsbruck, Innsbruck, Austria

**Keywords:** Autonomic dysfunction, Transthyretin amyloidosis, Autonomic testing, Tilt table testing, Orthostatic hypotension

## Abstract

**Background:**

Autonomic dysfunction is well recognized in hereditary transthyretin amyloidosis (ATTRv), but it has not been systematically studied in wild-type transthyretin amyloidosis (ATTRwt). Because ATTRwt primarily presents with cardiomyopathy, autonomic symptoms may mimic heart failure and lead to inappropriate treatment. Here we aimed to investigate the presence and extent of autonomic dysfunction in ATTRwt.

**Methods:**

In ATTRwt patients and controls, we performed an extensive autonomic examination, including standardized questionnaires, passive and active orthostatic challenges, Valsalva maneuver, deep breathing and sudomotor assessment.

**Results:**

20 ATTRwt patients and 20 controls were included. Composite Autonomic Symptom Score 31-scores were similar between the groups. Orthostatic challenges revealed impaired blood pressure (BP) and heart rate regulation in ATTRwt compared to controls (for passive orthostatic challenge: HR *p* = 0.001, systolic BP *p* = 0.010) and diastolic BP *p* = 0.006; for active orthostatic challenge: HR *p* = 0.001, systolic BP *p* = 0.002, diastolic BP *p* = 0.002). A lack of late phase 2 BP overshoot during Valsalva maneuver was observed in ATTRwt and Valsalva Ratio was pathological in 83% of ATTRwt versus 30% of controls (*p* = 0.020). The rate of pathological sweat tests did not differ between ATTRwt patients and controls.

**Conclusions:**

Autonomic symptoms in ATTRwt were infrequently reported. However, detailed assessment revealed cardiovascular autonomic dysfunction, which contributes to the overall clinical phenotype of ATTRwt.

## Introduction

Wild-type transthyretin amyloidosis (ATTRwt) is a rare multisystemic disease in which instability of tetrameric transthyretin proteins leads to deposition of transthyretin monomers in various tissues, including the myocardium and peripheral nervous system. Besides cardiomyopathy, progressive length-dependent peripheral (poly)neuropathy may be part of the clinical spectrum in a proportion of ATTRwt patients [1]. As unmyelinated fibers of the autonomic nervous system run within efferent and afferent somatic peripheral nerves, not only the large myelinated axons may be affected by amyloid deposits, but also smaller unmyelinated autonomic fibers. In patients with hereditary transthyretin amyloidosis (variant transthyretin amyloidosis, ATTRv), amyloid deposits can be found at different levels throughout the sympathetic nervous system and vagus nerve [2]. Autonomic symptoms in several genetic variants of ATTRv are common and clinically relevant [3]. Symptoms range from neurogenic orthostatic hypotension, dry eyes and mouth, bladder and sexual dysfunction to gastrointestinal dysmotility [3–5]. In ATTRv, symptoms of autonomic dysfunction are associated with quality of life impairment [3], and the development of autonomic dysfunction impacts morbidity and is considered a marker of disease progression [5]. Cardiovascular autonomic dysfunction might also carry an important prognostic value [6], as it may facilitate the development of life-threatening arrhythmias due to impaired cardiovascular autonomic control over the myocardial tissue [5]. Symptoms and signs of cardiovascular autonomic failure can closely resemble those of heart failure, including orthostatic hypotension, fatigue, exercise intolerance and syncope [7]. However, the underlying mechanisms and, crucially, the therapeutic approaches differ significantly between these conditions. For example, in heart failure, the typical therapeutic approach would consist of increasing neuro-humoral therapy (e.g., by use of beta-blockers) to counteract chronic excessive sympathetic activity and neurohormonal activation [8], while in cardiovascular autonomic failure, such an excessive blockade of the sympathetic nervous system may worsen orthostatic hypotension [9].

Due to the similarities in ATTRwt and ATTRv pathophysiology, autonomic dysfunction is likely to occur in ATTRwt as well, but this has not been systematically studied with standardized measures yet. As understanding and recognition of autonomic disturbances is important for patient therapeutic management, we performed a detailed study of autonomic nervous system dysfunction in a cohort of ATTRwt patients and age-matched healthy controls.

## Methods

### Patients

We prospectively recruited adult patients with ATTRwt cardiomyopathy managed at the cardiology outpatient clinic of the University Hospital of Innsbruck, Austria between November 2022 and November 2024. The diagnosis of ATTRwt was established in patients with a Perugini score > 1 on bone scintigraphy using 99mTc labeled 3,3 phosphone-1,2-DPD as tracer [10] and exclusion of free light chains in urine and serum, or in case of ambiguity (Perugini score = 1), presence of amyloid deposits in endomyocardial biopsy, with amyloid characterization performed by immunohistochemistry or mass spectrography. In all patients, absence of a *TTR* mutation was confirmed through *TTR* gene sequencing. Patients with vitamin B deficiencies, poorly controlled diabetes mellitus, significant carotid artery stenosis, severe coronary artery disease, hypertrophic cardiomyopathy with outflow tract narrowing and severe aortic or mitral valve stenosis and/or history of myocardial infarction were excluded.

For control purposes, we prospectively recruited an equal number of healthy individuals aged > 65 years, who fulfilled none of the exclusion criteria and, additionally, had no known neurologic or cardiac diseases. The study protocol was approved by the local ethics committee (Approval No: 1418/2021) and written informed consent was obtained from all patients. All procedures were performed in accordance with the 1964 Declaration of Helsinki, its later amendments, and the European Data Protection Regulation.

### Clinical evaluation

Clinical and demographic data were collected at baseline and integrated with information stored in the local medical electronic records. The extent of heart failure was classified using the New York Heart Association (NYHA) Functional Classification, based on limitations of physical activity. Disease duration was defined from the date of diagnosis to the date of neurological examination. Electrophysiological evidence for polyneuropathy was defined as the presence of abnormalities in at least two examined nerves. Standardized sensory and motor nerve conduction studies of the median and ulnar nerve, motor nerve conduction studies of the tibial and peroneal nerve, and sensory nerve conduction studies of the sural and superficial radial nerves were performed using a Dantec® Keypoint® Workstation, following our laboratory’s standard operating procedures and normative values.

### Patient-reported outcomes

The Composite Autonomic Symptom Score 31 (COMPASS 31) scale was used to assess autonomic dysfunction across six domains (range 0–100, higher scores indicating more severe impairment/symptoms), i.e., orthostatic intolerance, vasomotor, secretomotor, gastrointestinal, bladder and pupillomotor functions. The scores on the domains were calculated as per Sletten et al. [11]. Cut-off values to differentiate patients with and without autonomic dysfunction were derived from Singh et al., with a cutoff of 28.3 being applied for the sum score [12].

### Cardiovascular autonomic function assessment

Patients and healthy controls were instructed not to consume any caffeinated or taurine-containing beverages on the day of the examination, and to fast and abstain from nicotine consumption in the 2 h prior testing. Sympathomimetic, anticholinergic medication, and other cardio- and vasoactive drugs were paused for 48 hours before testing. Cardiovascular autonomic function assessments were performed in the morning hours. Orthostatic challenges were performed in a quiet setting with a constant room temperature (22 ± 1 °C). For the passive orthostatic challenge, we made use of a motorized tilt table. Heart rate (HR) and blood pressure (BP) were continuously monitored non-invasively with use of a Task Force® Monitor (CNSystems 2007). Measurements were performed after 10 min in the supine position for passive orthostatic challenges and after 5 min supine for active orthostatic challenges. The passive orthostatic challenge at an angle of 60° lasted 10 min, and the active orthostatic challenge lasted 5 min.

Classic orthostatic hypotension (OH) was defined as a sustained decrease of systolic BP ≥ 20 mmHg or diastolic BP ≥ 10 mmHg within three minutes of active or passive orthostatic challenge. Delayed OH was defined as a decrease of systolic BP ≥ 20 mmHg or diastolic BP ≥ 10 mmHg, measured five minutes after the active and/or 10 min after the passive orthostatic challenge [13]. Transient OH was defined as a systolic BP fall > 20 mmHg or diastolic > 10 mmHg within 15 s upon standing, and recovering within the first minute [14]. We excluded BP records that were considered artifacts, defined as systolic BP under 50 mmHg or over 250 mmHg, or diastolic BP under 30 mmHg or over 150 mmHg. In patients with ascertained OH, supine hypertension was defined as systolic BP ≥ 140 mmHg and/or diastolic BP ≥ 90 mmHg, after 5 min of rest in the supine position [15].

After active and passive orthostatic challenges, three trials of the Valsalva maneuver (15 s breath out in a mouthpiece against an expiratory pressure of 40 mmHg in a seated position) were performed to assess sympathetic outflow to the blood vessels and cardiovagal outflow to the heart. A Valsalva maneuver was excluded from analysis if the subject was not able to reach the expiratory pressure of 40 mmHg [16]. BP counter regulation during the Valsalva maneuver was registered and the BP overshoots during the late phase II and phase IV were used to appraise peripheral vascular sympathetic regulation [17]. These were calculated as follows: Phase II overshoot = mean arterial blood pressure (MAP) in the late phase II—MAP in the early phase II; Phase IV overshoot = MAP in the phase IV—MAP in the phase I [18].

For assessing cardiac parasympathetic outflow, the Valsalva ratio was calculated for the best performed Valsalva maneuver. It was calculated by dividing the highest HR in late phase II by the lowest HR in phase IV. Normative values for the Valsalva ratio were derived from Novak et al. [19]. The Valsalva ratio could not be calculated in patients with pacemakers or cardiac arrhythmias. Deep breathing tests were additionally performed, with participants instructed to deeply and slowly breath in and out at a pace of 6 breaths/minutes for one minute. The expiration/inspiration difference (E/I difference) was calculated as the average difference in heart rate between the end of expiration and the end of inspiration. Age- and gender adjusted normative values provided by Novak [19] were used to assess whether E/I differences were pathological.

### Sudomotor function assessment

Quantitative sudomotor axon reflex test (QSART) was performed on the right medial forearm (three fourths of the distance from the ulnar epicondyle to the pisiform bone), proximal leg (5 cm distal to the fibular head), distal leg (5 cm proximal to the medial malleolus) and feet (over the extensor digitorum brevis muscle) to assess C fiber function. The skin was stimulated by iontophoresis of 10% acetylcholine solution for 5 min. The sweat volume response was recorded with a high precision hygrometer and adjusted per age according to manufacturer reference values (Q-SWEAT, WR-Medicals, Maplewood, USA).

The degree of abnormality was assessed as follows: mildly abnormal in patients with a single site abnormality and > 50% of lower limit or length-dependent pattern or persistent sweat activity; moderate abnormal, single site < 50% of lower limit or two or more sites reduced and > 50% of lower limit; severely abnormal, in patients with two or more sites < 50% of lower limit. Normative values were provided by Novak [19].

Sudomotor function was also assessed by testing the sympathetic skin response (SSR), recorded with surface electrodes from the right hand and right foot. Stimuli to elicit the SSR were applied to the median nerve at an intensity of 25 mA and a duration of 0.2 ms with use of a Dantec® Keypoint® electrical stimulator.

### Statistical analysis

The patient characteristics were summarized using descriptive statistics. The data distribution was assessed using Shapiro Wilk tests and visually using histograms and Q–Q plots. Data is shown as mean and standard deviation (SD) for normally distributed variables and additionally median and interquartile range for non-normally distributed variables, and range, when appropriate. Differences between groups were investigated with Student’s *t* test for independent samples or Mann–Whitney *U* test, according to their distribution. Chi-squared tests and *Z* test for proportions were used to compare frequencies. Repeated-measures ANOVA was used to compare passive and active orthostatic challenge parameters between ATTRwt patients and healthy controls, prioritizing the analysis of heart rate and blood pressure behavior across all time points over pairwise comparisons at single timepoints.

Statistical analysis was performed using SPSS version 29.0.0 (IBM Corp, Armonk, USA) and GraphPad Prism version 9.4.1 for Windows (Graphpad Software, Boston, Massachusetts USA). Due to the explorative nature of our study, no correction for multiple comparisons was applied.

## Results

Twenty patients with ATTRwt and twenty age-matched healthy controls were included. Clinical and demographic data are reported in Table 1. The mean age at the timepoint of examination in the ATTRwt cohort was 78.0 ± 4.7 years (range 66–84) and the majority (85%) of patients were male. Thirteen ATTRwt patients had cardiovascular comorbidities (*n* = 12 arterial hypertension, *n* = 6 atrial fibrillation, *n* = 7 non-severe coronary artery disease, *n* = 2 mild heart valve stenosis). Five ATTRwt patients were under levothyroxine treatment and had stable thyroid function parameters and 4 patients had well-controlled diabetes mellitus. The healthy controls had none of these comorbidities. In the ATTRwt cohort, 5 patients were treatment-naïve, 12 patients were treated with tafamidis and 3 patients were in a pharmacological study additionally receiving either eplontersen or placebo.

**Table 1:** Clinical and demographic data for each patient and the whole cohort

	ATTRwt patients (*n* = 20)	Healthy controls (*n* = 20)	*p* value
Age at examination, y	78.0 ± 4.7	75.1 ± 3.0	0.052
Disease duration, m	21.1 ± 27.0 10.5 (IQR 4.0–29.5)	–	–
BMI (kg/m^2^)	25.2 ± 3.0 24.9 (IQR 23.3–27.1)	23.9 ± 3.7 23.5 (IQR 21.9–26.5)	0.201
Women, *n* (%)	3 (15%)	7 (35%)	** < 0.05**
Polyneuropathy, *n* (%)	16 (80%)	10 (50%)	** < 0.05**
Use of vasoactive drugs*	14 (70%)	6 (30%)	** < 0.05**
NYHA classification			
1	2	20	** < 0.01**
2	17	0	** < 0.01**
3	1	0	^⁋^
4	0	0	^⁋^
Atrial fibrillation	6 (30%)	0 (0%)	** < 0.01**
Cardiac pacemaker	8 (40%)	0 (0%)	** < 0.01**

Bold values indicate statistical significance (*p* < 0.05)

Data is represented as mean ± standard deviation and supplemented with median with 95% confidence interval, according to their distribution

^*^Vaso-active drugs were paused for at least 48 h before examination. ^⁋^Subgroup sample size too small for statistical analysis

*BMI* body mass index; *IQR* interquartile range; *m* months; *NYHA* New York Heart Association classification; *y* years

### Patient-reported outcomes

**Fig. 1:**
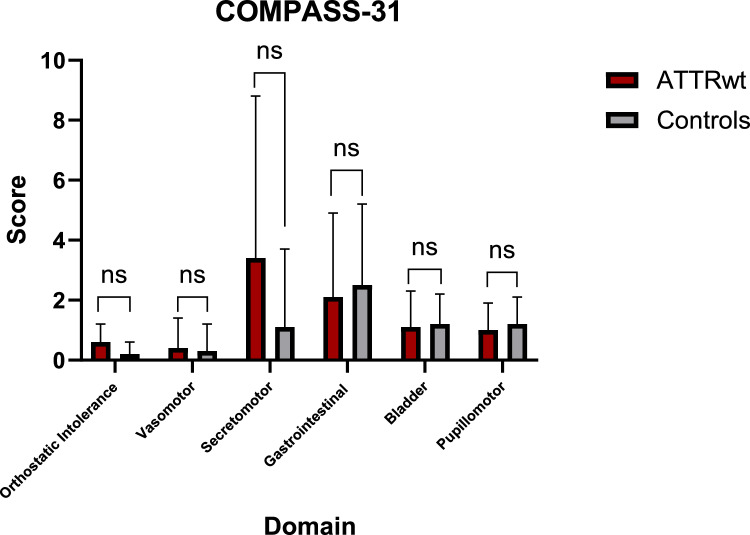
Patient-reported symptoms of autonomic dysfunction. Data are represented as mean and error bars represent standard deviation. *COMPASS 31* Composite Autonomic Symptom Score 31; *ATTRwt* wild-type transthyretin amyloidosis; *ns* not significant

In the COMPASS 31 questionnaire, one ATTRwt patient achieved a sum score above the cut-off value of 28.3, while none of the controls did. COMPASS 31 total scores did not differ significantly between ATTRwt patients and healthy controls (8.6 ± 0.9 versus 6.4 ± 4.7, *p* = 0.394), as did not subdomain scores (see Fig. 1). In both the ATTRwt cohort and the healthy cohort, there was no significant difference in score between patients with and without polyneuropathy (*p* = 0.070 and *p* = 0.151, respectively).

### Orthostatic testing

In one of the ATTRwt patients, an active orthostatic challenge test was not performed because of presyncope during the passive orthostatic challenge due to OH. In total, thirteen ATTRwt patients and 12 healthy controls fulfilled the criteria for OH (any form). Two (10%) ATTRwt patients fulfilled the criteria for classic OH, while none of the healthy controls met these criteria. Delayed OH according to the criteria was found in 5 (25%) ATTRwt patients and 6 (30%) healthy controls (*p* = 0.723). Transient OH was found in 9 (45%) ATTRwt patients and 11 (55%) healthy controls (*p* = 0.527).

**Fig. 2:**
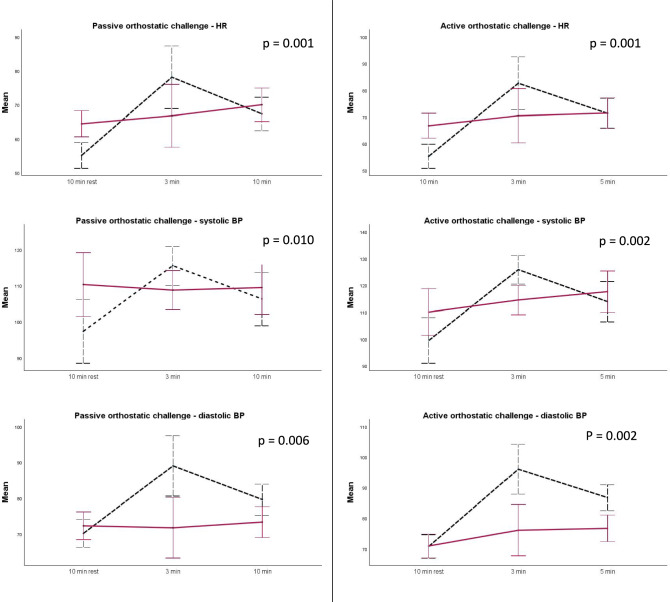
ANOVA for repeated HR and BP measurements during passive and active orthostatic challenges in patients with ATTRwt amyloidosis versus healthy controls. Error bars indicate 95% confidence interval. Red continuous line = ATTRwt patients, black dotted line = healthy controls. *ATTRwt* wild-type transthyretin amyloidosis; *BP* blood pressure; *HR* heart rate

In both the passive and active orthostatic challenges, we found that ATTRwt patients had a significantly smaller BP and heart rate increase than healthy controls (see Fig. 2). Mean systolic BP in supine position was significantly higher in the ATTRwt cohort compared to healthy controls (*p* < 0.001), but none of the ATTRwt patients or healthy controls fulfilled the criteria for supine hypertension.

A sensitivity analysis was performed, in which active and passive tilt table results were compared among ATTRwt patients with atrial fibrillation and/or cardiac pacemakers versus ATTRwt patients without. For the passive tilt table test, systolic BP, diastolic BP and heart rate were comparable among the groups (*F* = 1.132, *p* = 0.316, *F* = 2.680, *p* = 0.104, and *F* = 0.262, *p* = 0.771, respectively). No difference was seen in the active tilt table test either (*F* = 0.509 *p* = 0.534, *F* = 0.076, *p* = 0.895 and *F* = 0.342, *p* = 0.633, for systolic and diastolic BP, and heart rate respectively).

### Valsalva maneuver

Significantly more ATTRwt patients (65%) showed a lack of BP overshoot in late phase 2 as compared to healthy controls (30%) (*p* = 0.026). This was not seen at phase 4, as 75% of ATTRwt patients and 70% of healthy controls showed a lack of BP overshoot (*p* = 0.723).

Valsalva ratio could not be calculated in 14 ATTRwt patients due to cardiac pacemaker implantation or atrial fibrillation. Among the remaining patients, 5 out of 6 (83%) showed a pathological Valsalva ratio versus 6 out of 20 healthy controls (30%, *p* = 0.020).

When comparing within the patient cohort, the frequency of missing blood pressure (BP) overshoots during late phases 2 and phase 4 of the Valsalva maneuver did not differ significantly between those with and without pacemaker or atrial fibrillation (*p* = 0.052 and *p* = 0.573, respectively). Patients with AF/pacemaker were significantly older than patients without (80 versus 74 years, *p* = 0.012).

### Deep breathing

Using age- and gender adjusted normative values, the E/I difference was pathological in 67% of ATTRwt patients and 47% of healthy controls (*p* = 0.408).

### Sudomotor function assessment

QSART was performed in all healthy controls, while three ATTRwt patients did not consent for QSART due to hypersensitive skin. 53 percent of ATTRwt patients and 40% of healthy controls had moderate or severe abnormalities in QSART testing, *p* = 0.431 (see Table 2).

**Table 2:** Degree of abnormality in QSART findings in ATTRwt patients and healthy controls

Degree of abnormality	ATTRwt*	Healthy controls	*p* value
Number of patients	17 (100%)	20 (100%)	
Normal/mild abnormal	8 (47%)	12 (60%)	
Moderate/severe abnormal	9 (53%)	8 (40%)	0.431

Adopted from Novak et al. [11]

^*^3 patients did not consent for QSART and were excluded from this analysis

*ATTRwt* wild-type transthyretin amyloidosis; *QSART* quantitative sudomotor axon reflex test

In all healthy controls with severe QSART abnormalities (*n* = 8) and in 3 of 4 ATTRwt patients with severe abnormalities in QSART, we also found electrophysiological evidence of large fiber polyneuropathy.

SSR was measured in all patients and all controls. The SSR was absent in the lower extremities of five and in the upper extremities of one ATTRwt patient. Among healthy controls, absence was observed in one upper and one lower extremity. The frequency of absent SSRs did not differ significantly between groups (*p* = 0.113). All ATTRwt patients with absent SSRs also had pathological QSART results.

## Discussion

Here we performed a detailed assessment of cardiovascular autonomic and sudomotor function in ATTRwt patients and in healthy controls, while controlling for potential confounders, including age-related changes and medication effects. Our study revealed autonomic dysfunction in patients with ATTRwt, particularly affecting cardiac parasympathetic dysfunction and sympathetic adrenergic dysfunction, which remained largely asymptomatic in the majority of patients at screening questionnaires. Peripheral nervous system involvement has been increasingly recognized as part of the clinical spectrum in ATTRwt [20, 21], and the presence of autonomic dysfunction suggests a broader impact on the nervous system than previously described.

In the COMPASS 31 questionnaire, the overall frequency of autonomic symptoms was similar between individuals with ATTRwt and healthy controls. It is, however, important to recognize that COMPASS 31 does not specifically assess symptoms of neurogenic origin. Instead, it captures a broad spectrum of autonomic-related complaints, many of which are common in the elderly population and may arise from both neurogenic and non-neurogenic causes. Although the COMPASS 31 has been widely used and validated in older populations, its clinimetric properties may be in fact influenced by the occurrence of cardiac and other comorbidities [11, 22]. Beyond this, the mismatch between signs and symptoms of OH is a recognized phenomenon even in primary autonomic disorders such as Parkinson’s disease. In these patients, it has been shown that the lowest blood pressure levels reached in the upright position rather than the amplitude of the blood pressure fall (on which a diagnosis of OH is based), may better predict the presence of orthostatic symptoms, especially if the blood pressure falls below the threshold for cerebral autoregulation [23].

While symptoms of autonomic dysfunction may remain unspecific or even absent, detailed assessment of autonomic function unveiled several cardiovascular autonomic deficits in our ATTRwt cohort. Compared to healthy controls, ATTRwt patients exhibited cardiac parasympathetic dysfunction, evidenced by impaired heart rate modulation during the Valsalva maneuver and, to a lesser extent, during deep breathing. While the degree of cardiovagal involvement may vary among individuals with ATTRwt, its presence carries significant prognostic implications. The cardiovagal outflow is critical for modulating heart rate and myocardial contractility in response to blood pressure fluctuations, and unrestrained sympathetic activity has been associated with elevated cardiovascular risk, independently of average BP levels [24–26]. In patients with heart failure, the lack of vagally mediated negative chronotropic and inotropic effects leads to sustained sympathetic stimulation of cardiac adrenergic receptors, ultimately worsening the underlying heart failure [8].

The normal response to orthostatic challenge tests is a compensatory heart rate increase and an activation of the sympathetic system, which induces arterial and venous vasoconstriction to stabilize the BP [27]. In individuals with OH, this response is impaired. The prevalence of OH increases with age, affecting up to 22% of healthy individuals aged 60 and older [28]. Changes in the autonomic nervous system and reduced baroreceptor sensitivity are associated with normal aging [29, 30], leading to the loss of appropriate compensatory mechanisms to prevent orthostasis. Additionally, non-neurogenic factors common in older adults, such as volume depletion, polypharmacy, and cardiovascular aging [27, 30, 31], can negatively impact BP regulation in response to positional changes.

While overt OH was not frequent in our ATTRwt cohort, detailed assessment under continuous non-invasive hemodynamic monitoring revealed significant sympathetic adrenergic dysfunction compared to healthy controls. ATTRwt patients indeed showed impaired heart rate and BP regulation during both passive and active orthostatic challenges. The typical blood pressure overshoot during phase II of the Valsalva maneuver, another index of sympathetic noradrenergic control over peripheral blood vessels, was also frequently missing, altogether indicating a degree of sympathetic autonomic impairment beyond that expected from normal aging [19].

In ATTRv, electron microscopy has demonstrated direct amyloid fibril–induced damage to Schwann cells and loss of both myelinated and unmyelinated nerve fibers [32, 33]. The resulting sensory–motor polyneuropathy follows a length-dependent pattern [34], and a similar pattern is expected in autonomic neuropathy. Given that the vagus nerve is the longest autonomic nerve in the body, cardiovagal dysfunction can be expected as an earlier manifestation of autonomic neuropathy, while sympathetic denervation may developed later on as the disease progresses [35].

We did not observe differences in postganglionic sympathetic cholinergic sudomotor function using QSART and SSR between ATTRwt patients and healthy controls. This observation indicates that factors beyond nerve length, such as the type of neurotransmission, specifically noradrenergic versus cholinergic, may play a role in the vulnerability of nerves in developing sympathetic dysfunction. Ongoing histopathological studies of skin biopsies from individuals with ATTRwt has the potential to unravel whether ATTRwt preferentially affects noradrenergic rather than cholinergic postganglionic sympathetic fibers.

To prevent inappropriate initiation or escalation of pharmacological therapy, it is essential to differentiate cardiac autonomic dysfunction from symptoms resulting from cardiac failure. Symptoms, such as orthostatic dizziness, dyspnea, and fatigue, can be easily misinterpreted as signs of cardiac decompensation in individuals with ATTRwt amyloidosis. This misinterpretation may lead to inappropriate escalation of heart failure treatment, which can further aggravate the underlying orthostatic BP dysregulation. In cases with significant cardiovascular autonomic dysfunction, pharmacological neuro-humoral inhibition (e.g., using beta-blockers or RAAS inhibitors) should be used cautiously or avoided altogether. When such therapies are indicated for heart failure, reduced doses may be required to prevent worsening of autonomic symptoms, as excessive sympathetic blockade can exacerbate orthostatic intolerance [36]. Nighttime dosing of antihypertensive medications has also been proposed to reduce daytime hypotension and mitigate the risk of falls [37, 38]. This strategy may be particularly relevant in ATTRwt patients, who in our study exhibited higher supine BP values and may be therefore more susceptible to nocturnal hypertension, as previous work showed that pathological nocturnal BP profiles are associated with daytime supine hypertension and altered BP responses during Valsalva maneuvers [18].

Ultimately, an individualized risk–benefit assessment is needed to balance the benefits of neuro-humoral inhibition for heart failure against its potential to impair BP regulation upon standing. Similarly, non-pharmacological approaches to orthostatic hypotension that expand plasma volume (e.g., increased sodium and water intake), should be implemented with caution in patients with cardiac comorbidities. Pharmacological measures for orthostatic hypotension, including sympathomimetic agents (midodrine, etilefrine, droxidopa) and fludrocortisone, should likewise be reserved for cases that remain refractory to non-pharmacologic measures, and patients carefully monitored at follow-up for exacerbation of heart failure [39].

This study has limitations. The presence of cardiomyopathy in itself, and the corresponding pharmacological therapy in ATTRwt patients, as well as the occurrence of atrial fibrillation and other rhythm disorders requiring pacemakers in some of them, are risk factors for (exacerbating) orthostatic hypotension [40–42], and all may have influenced the results of cardiovascular autonomic function testing. To appraise the influence of cardiac comorbidities, a sensitivity analysis was performed, and this did not pinpoint diverging cardiovascular autonomic profiles in ATTRwt patients with or without atrial fibrillation or pacemakers. Abnormalities in BP counter regulation during the Valsalva maneuver have also been observed, reflecting impaired sympathetic control of peripheral vessels that are rather independent of cardiac function. This suggest that the observed impairment in heart rate and BP regulation likely resulted from a combination of heart failure and baroreflex dysfunction. Conduction of all cardiovascular autonomic function tests after withholding vasoactive medications further helped to minimize a key non-neurogenic confounder. For future studies, a control group with non-amyloid cardiomyopathy could be utilized to distinguish the effects of amyloid deposition from those of common cardiac comorbidities on autonomic dysfunction. Although the statistical validity of this study is limited by its sample size, this must be considered in context of the disease’s rarity. Notably, this study is the first to provide a detailed autonomic phenotyping of patients with ATTRwt.

In conclusion, our findings suggest that autonomic dysfunction is an underrecognized component of the neuropathy spectrum in ATTRwt. Given that peripheral neuropathy is generally milder in ATTRwt than in ATTRv, autonomic involvement is also expected to be less pronounced and may be further modulated by age- and amyloidosis-related cardiovascular changes [27]. Especially in ATTRwt, where autonomic dysfunction and cardiac failure often coexist, early and accurate identification of autonomic impairment using standardized autonomic testing is essential to warrant its identification and guide an appropriate management.

## Data Availability

The data that support the findings of this study are available from the first author, VK, upon reasonable request and permission from the responsible bodies.
